# Low intensity surface fire instigates movement by adults of *Calosoma frigidum* (Coleoptera, Carabidae)

**DOI:** 10.3897/zookeys.147.2084

**Published:** 2011-11-16

**Authors:** Jenna M. Jacobs, J. A. Colin Bergeron, Timothy T. Work, John R. Spence

**Affiliations:** 1 Département des Sciences Biologiques, Université du Québec à Montréal, Pavillon des sciences biologiques (SB), 141 Avenue du Président-Kennedy Montréal (Québec), H2X 1Y4, Canada; 2 Department of Renewable Resources, University of Alberta, 751 General Services Building, Edmonton, Alberta, T6G 2H1, Canada

**Keywords:** *Calosoma*, foraging, behavior, prescribed fire, defoliation

## Abstract

The genus *Calosoma* (Coleoptera: Carabidae) is a group of large, sometimes ornate beetles, which often voraciously attack caterpillars. Many studies have reported *Calosoma* beetles being highly conspicuous during defoliator outbreaks. Based on observations of individual beetle behavior, patterns of activity density and phenology we provide a hypothesis on how environmental cues may synchronize *Calosoma* activity with periods of high defoliation. We have observed that adults of *Calosoma frigidum* construct underground burrows similar to those reported to be created by larvae for pupation. We propose that small increases in soil surface temperature caused either by defoliation events or decreased albedo of blackened, burned soil causes beetles to leave their underground burrows and begin foraging. Indirect support for this hypothesis comes from high levels of adult *Calosoma frigidum* collected in relatively small patches of burned forest (<200m^2^) relative to the surrounding mosaic of unburned forest shortly after a prescribed surface burn.

## Introduction

*Calosoma frigidum* Kirby, also known as “The Cold Country Caterpillar Hunter” (Erwin 2007) has long been of interest to entomologists because of its voracious appetite for caterpillars (Burgess and Collins 1917). Adults are macropterous, good flyers and frequently climb trees to feed on caterpillars (Lindroth 1961). Larvae are known live on the ground and feed voraciously on lepidopteran pupae and larvae that have dropped to the ground (Larochelle and Larivière 2003; Lindroth 1961). Adults and larvae *Calosoma frigidum* feed on a variety of defoliator species including satin moth (Wagner and Leonard 1980), bruce spanworm (Crins 1980), gypsy moth (Cameron and Reeves 1990), spruce budworm (Sanders and Frankenhuyzen 1979) and forest tent caterpillar (Parry et al. 1997), and can become highly conspicuous during outbreak periods (Cameron and Reeves 1990; Jacobs et al. 2008; Loesh 1977).

Prey density has been linked to *Calosoma* ovarian development, oviposition behaviour, and female activity (Jeffords and Case 1987; Spieles and Horn 1998; Weseloh 1993). Weseloh (1985) observed that populations of the introduced biological control agent, *Calosoma sycophanta*, were high in years following collapse of lepidopteran outbreaks, however individuals were not active nor dispersing. Weseloh (1993) further found that few females would reproduce unless fed caterpillars in the first week after ending diapause, and furthermore, it is known that some adults remain dormant in overwintering soil cells all summer when moth numbers are low (Vasik 1972). This behavior may allow beetles to remain in an extended dormancy over multiple years when prey populations are low. Despite the obvious link between populations of *Calosoma* beetles and their prey, it remains unclear what signals trigger or inhibit activity of these caterpillar hunters.

Several studies have noted increased abundance of *Calosoma* beetles in pitfall traps in response to changes in habitat variables. Jacobs et al. (2008), for example, observed increased catch rates of *Calosoma frigidum* after low levels of forest harvest, however catch rates declined when forest harvest exceeded 50% intensity. Koivula et al. (2006) found a decline in catch rates with increased salvage harvesting. Other authors have shown that activity-density of*Calosoma frigidum* along with that of a closely related species, *Calosoma calidum* (Fabricius), was positively impacted by burning, salvage harvesting and a herbicide treatment applied after harvesting, however catches of both these species in control stands were extremely low (Cobb et al. 2007).

Based on comparisons of pitfall trap catch rates *Calosoma frigidum* in a mosaic of burned and unburned forest, we hypothesize that activity of *Calosoma frigidum* is triggered by increased soil temperature. We explain that adult *Calosoma* construct an underground burrow, similar to that made for pupation (Larochelle and Larivière 2003) and documented for other *Calosoma* species (Vasik 1972), and suggest that they emerge from the burrow in response to soil warming caused by severe defoliation events. Alternatively, as we show here, activity may be increased when temperature is raised by the albedo of burned, blackened soil.

## Methods

### Study Site

This study took place at the Ecosystem Management by Emulating Natural Disturbance (EMEND) research site in northwestern Alberta, Canada (56°46’13”N, 118° 22’28”W). The 10 ha stand chosen for the study was dominated by trembling aspen (*Populus tremuloides* Michx.) and balsam poplar (*Populus balsamifera* L.) with the understory dominated by green alder (*Alnus crispa* (Aiton))and river alder(*Alnus tenufolia* Nuttall).  Further information on the EMEND site and ground beetles responses can be found in Work et al. (2010, 2004) and Jacobs et al. (2008).

On April 26^th^, 2000 a low intensity prescribed burn using drip torches was initiated as an EMEND treatment. The severity of the fire was not sufficient to kill trees and by the end of May evidence of fire was limited to some superficial charcoal deposits on the forest floor and at the base of trees. Surveys of the stand following fire revealed a mosaic of patches where fire had consumed a small portion of the forest floor and scorched tree bases adjacent to patches with no evidence of fire.

Within the stand subjected to prescribed burning we selected four areas (each ca. 100m^2^) in which burning was clearly evident and four additional areas with no evidence of burning, and installed two pitfall traps per area (16 in total) on June 5^th^, 2000. Our pitfall traps consisted of an outer permanent (1L) cup, acting as a sleeve to maintain the pit and minimize disturbance at checks, and a removable (500ml) inner cup (Spence and Niemelä 1994). Traps were filled with 200ml of silicate-free ethylene glycol as a killing agent and preservative and were covered with an elevated 10cm × 10cm wood roof. Arthropods captured in the traps were collected every 2–3 weeks until August 16^th^ 2000, and the sites were similarly resampled May 9^th^ to August 20^th^ 2001. Samples were stored in 75% ethanol and all beetles in the samples were identified to the level of species.

In an effort to make general observations of the beetle, we examined five adult *Calosoma frigidum* in the lab in 5L glass jars, filled with about 10cm of soil. These jars were left at room temperature for approximately 2 years.

### Data Analysis

Community level analysis revealed virtually no effect of burning on the larger beetle community or on activity of any individual species except for *Calosoma frigidum*. We therefore restricted the scope of this study to explaining the response of this species to the EMEND burning treatment. All analyses were conducted in R 2.12.1 (R Development Core Team 2010). Differences in *Calosoma frigidum* catch rates between treatments and across collection dates was analyzed using a two factor repeated measures analysis of variance (RM-ANOVA) using the aov function. Tukey HSD post-hoc tests were carried out using the HSD.test function in the agricolae library of R (Felipe de Mendiburu 2009).

## Results

**Figure 1.**
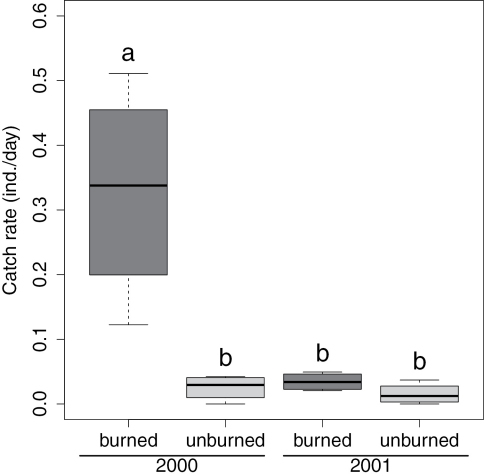
Box and whisker plot of catch rates of *Calosoma frigidum* in burned and unburned patches. Letters denote the results of a Tukey’s HSD post-hoc test following a 2 factor repeated measures ANOVA. Box represents the upper and lower quartiles divided by the median and whiskers are the largest and smallest values.

**Figure 2.**
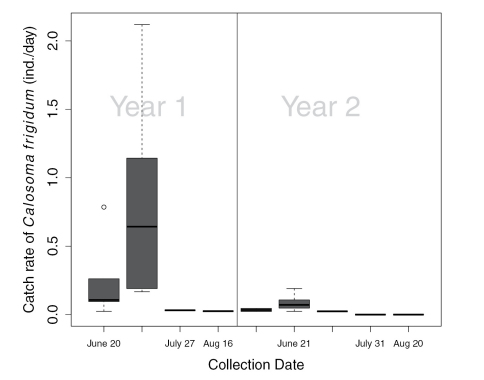
Box and whisker plot of catch rates in burned patches, by collection date, for the year of the fire (year 1) and the following year (year 2). Box represents the upper and lower quartiles divided by the median and whiskers are the largest and smallest values, excluding outliers represented by circles ○.

We collected 298 individuals of *Calosoma frigidum*, representing 14% of the total catch of 74 species. Catch rates of *Calosoma frigidum* were significantly higher in the burned patches during the year of the fire (2000) than in the unburned patches or anywhere in the stand the following year (Figure 1, RM-ANOVA, Treat x Year, F_1,6_=0.08, P=0.017). The highest catch rates were recorded in the July 11^th^ collection in 2000 and for the June 21 in 2001 (Figure 2). However, despite similarity in activity pattern, the overall activity in 2000 was much higher than in the following year.

In the laboratory one of the adult beetles created a burrow ca. 4cm under the soil surface as the soil dried. Whether the other beetles brought into the lab also subsequently created these burrows is unknown, as this behavior was not specifically under study.

## Discussion

Increased catch rates of *Calosoma frigidum* observed in the same year as the prescribed burn could be explained by either increased activity in burned plots or actual differences in population density between burned and unburned areas. While we are not capable of completely discounting this second explanation, we favor the first for several reasons. First, our study site was very homogeneous and the chances of all four burned patches being located in areas of high *Calosoma frigidum* density and all four unburned patches in areas of low *Calosoma frigidum* density seems low. Second, we observed increases in catch rates within the same year as the fire, discounting the possibility of increases due to reproduction. Thirdly, we feel it is unlikely that local densities increased as a result of immigration of *Calosoma frigidum* into the burned areas for the following reasons. If local density increased, the 3–5 year life span (Larochelle and Larivière 2003) of this species would suggest that we should observe continued elevated activity-density in the years following the fire. Furthermore, if immigrants were attracted to the burn, we would also expect to see higher activity-density patterns in surrounding unburned areas during this period. Also, in a concurrent study on wood-associated beetles using flight traps, we collected just 9 individuals of *Calosoma frigidum* with no patterns among treatments or between years. Together these three points suggest that the observed increases in catch rates are a function of increased activity rather than highly localized differences in density due to either reproduction or immigration.

We propose that the fire event itself had little direct influence on the beetles because it would have been of very short duration. Instead we posit that charcoal deposited on the soil surface in the wake of the fire resulted in higher soil temperature from decreased surface albedo. Sustained increases in soil temperatures from sun exposure in burned sites may be similar to those during severe defoliation and could serve as a signal to adult beetles to begin foraging when potential prey populations are high. This could explain the results of Cobb et al. (2007) who found high activity levels of both *Calosoma frigidum* and *Calosoma calidum* following an herbicide treatment on a young regenerating conifer stand. Under our hypothesis, removal of the dense shrub layer resulted in small increases in soil temperature prompting movement by these caterpillar predators.

Decreasing forest cover caused by forest harvest could also be associated with small increases in temperature of the soil surface, explaining why catch rates of *Calosoma frigidum* increased in residual strips compared to nearby dense forests in Maine (Jennings et al. 1986). This would also explain why the highest levels of capture were recorded at 50% green tree retention the 2 years following harvest at EMEND (Jacobs et al. 2008). Although it appears that small increases in the temperature of the soil surface could increase the activity of *Calosoma frigidum*, results from EMEND suggest that the explanation cannot be simple. Clearly, the complete lack of trees after more intensive harvest was associated with cessation of activity by this beetle or perhaps with dispersal by flight. Retention levels below 50%, quickly caused catch rates of *Calosoma frigidum* to drop (Jacobs et al. (2008). Similarly, Koivula and Spence (2006) initially recorded high catch rates of *Calosoma frigidum* following fire, and noted decreased catch rates as the intensity of salvage harvesting increased.

The capacity of adult beetles to construct underground burrows in conjunction with their long life span may permit *Calosoma* to effectively exploit cyclic defoliator populations characteristic of the boreal zone (e.g., those of forest tent caterpillar, bruce spanworm and spruce budworm). *Calosoma* populations could initially build-up during an outbreak through a combination of both localized immigration from neighboring stands and emergence of resident adults from underground burrows within the stand. If defoliator populations remain high, both immigrants and residents could conceivably accumulate in the stand in adult burrows in the soil. When an outbreak collapses and there are no signals to emerge and run, these adults could lay dormant for several years. As the next outbreak unfolds, increased soil temperatures would effectively synchronize adult beetles with an increasing source of prey as well with potential viable congeners to facilitate reproduction. The proportions of these individuals that emigrate following the collapse or remain in adult burrows as a ‘sit and wait’ strategy should vary with outbreak frequency, but such data remain to be collected.

## Conclusions

We have offered a possible mechanism for initiating the foraging response in dormant *Calosoma* beetles, using specific evidence from *Calosoma frigidum* in NW Alberta, Canada. Increases in the catch rate and activity patterns of *Calosoma* beetles have been recorded during defoliation events, herbicide treatments, mechanical canopy thinning and soil charcoal deposits following fire. All these events have the potential to warm the soil surface in a sustained way, and we suggest that such warming could cue beetles to emerge from their underground burrows and begin foraging. Although we acknowledge that this evidence is somewhat circumstantial, we hope that it offers useful insight to inspire further work about what triggers of foraging behavior in this very interesting beetle.
